# Intelligent Fault Diagnosis of Delta 3D Printers Using Attitude Sensors Based on Support Vector Machines

**DOI:** 10.3390/s18041298

**Published:** 2018-04-23

**Authors:** Kun He, Zhijun Yang, Yun Bai, Jianyu Long, Chuan Li

**Affiliations:** 1 School of Electromechanical Engineering, Guangdong University of Technology, Guangzhou 510006, China; hekun@dgut.edu.cn (K.H.); yangzj@gdut.edu.cn (Z.Y.); 2 School of Mechanical Engineering, Dongguan University of Technology, Dongguan 523808, China; baiyun@dgut.edu.cn (Y.B.); longjy@dgut.edu.cn (J.L.)

**Keywords:** delta 3D printer, fault diagnosis, attitude sensor, support vector machine, condition monitoring

## Abstract

Health condition is a vital factor affecting printing quality for a 3D printer. In this work, an attitude monitoring approach is proposed to diagnose the fault of the delta 3D printer using support vector machines (SVM). An attitude sensor was mounted on the moving platform of the printer to monitor its 3-axial attitude angle, angular velocity, vibratory acceleration and magnetic field intensity. The attitude data of the working printer were collected under different conditions involving 12 fault types and a normal condition. The collected data were analyzed for diagnosing the health condition. To this end, the combination of binary classification, one-against-one with least-square SVM, was adopted for fault diagnosis modelling by using all channels of attitude monitoring data in the experiment. For comparison, each one channel of the attitude monitoring data was employed for model training and testing. On the other hand, a back propagation neural network (BPNN) was also applied to diagnose fault using the same data. The best fault diagnosis accuracy (94.44%) was obtained when all channels of the attitude monitoring data were used with SVM modelling. The results indicate that the attitude monitoring with SVM is an effective method for the fault diagnosis of delta 3D printers.

## 1. Introduction

Additive manufacturing, also known as 3D printing [1], is a manufacturing technique based on the idea of material deposition and its curing layer by layer which can be implemented through different ways and materials [2]. Since it has a lot of merits compared to traditional manufacturing methods, the applications of the 3D printing have dramatically increased in the last few decades [3]. The primary advantage of the 3D printing is the ability to create almost any shape or geometric feature [4]. However, one of the main disadvantages is its inferior dimensional accuracy, since the precision of the 3D printing is influenced by many factors which have seriously restricted its sustainable development [5]. One of the most important factors is the mechanical transmission of the 3D printer. Hence it is necessary to monitor the transmission condition of the 3D printer even if it has precision components [6].

In general, 3D printers can be divided into serial structure ones and parallel structure ones [7]. Although serial mechanisms with open kinematic structures usually provide large workspace, parallel mechanisms that have closed kinematic chains show such advantages as low weight, compact structure and especially good stiffness [8]. As one of the most common and basic parallel devices, delta 3D printers are employed as the research object of the fault diagnosis in this work. For parallel mechanisms, kinematics [9], dynamics [10], joint clearance [11] and friction [12] models have been well studied. In [13], inverse and forward kinematic analyses were performed to design a parallel robotic leg. An optimal parameter was obtained via a multi-objective optimization. In [14], a parallel manipulator applied for automobile piece testing purposes was designed through the analyses of kinematics, singularities and dynamics. However, even well-designed 3D printers may encounter faults during their lifetime. This will influence the quality of the printed products [15]. From the viewpoint of mechanism, delta 3D printers are more likely to suffer faults than serial mechanism ones due to their more complicated transmission structures [16]. For this reason, early diagnosis of incipient faults is a feasible way to provide motion compensation and to avoid economic losses for delta 3D printers.

Different signals may be collected from machinery to analyze their health condition [17]. Vibratory [18,19], electric [20], oil-based [21], thermal [22], and acoustic [23] signals have all been used to diagnose faults of machinery or its components. To improve bearing defect detection performance, Li et al. [24] proposed a criterion fusion approach to guide the spectral segmentation process with vibration signals. In [25], a fault diagnosis filter is designed for a nonlinear discrete-time system in the Takagi-Sugeno fuzzy form with faults and unknown inputs. To enhance fault detection for gearboxes, Jing et al. [26] introduced an approach based on multi-sensor data fusion with deep convolutional neural networks. All these techniques have made great contributions to the fault diagnosis field, but they may not be completely applicable to delta 3D printers. Hence, this paper suggests an attitude monitoring method incorporating a machine leaning algorithm for the fault diagnosis of the delta 3D printer.

There are two basic steps for diagnosing the delta 3D printer in the present method. The first one is the data acquisition from the attitude sensor mounted on the moving platform of the printer. Attitude sensors have been widely applied in various fields such as to balance robots, to stabilize cameras and devices to analyze human movement. The attitude sensor can be made extremely light and compact by using micro-electro-mechanical system (MEMS) technology [27]. The attitude sensor based on MEMS usually consists of three-axial gyros (angular velocity signals), three-axial accelerometers (vibratory acceleration signals) and three-axial magnetometers (magnetic field intensity signals) [28]. Data from all sensing elements can be fully utilized by a Kalman filter [29], and further to obtain attitude angles relative to the reference frame of the attitude sensor including pitch, roll and yaw. At the same time, the raw data of sensing elements are also obtained [30]. The attitude of the moving platform can be reflected in these data. The second step in the present method is to obtain the health condition using data classification by an intelligent algorithm [31,32]. In [33], the approach based on a SVM classifier with a multi-sensory system is proposed to diagnose wind turbines. In [34], multimodal deep support vector classification with homologous features was applied to diagnose gearboxes, and it achieved the best fault classification rate compared to representative deep and shallow learning methods. In [35], fault diagnosis is performed for the gearbox of wind turbines by using deep neural networks, which provided a classification accuracy of 98%. SVM can be applied as a pattern classification technique proposed by Vapnik in 1995 [36]. Suykens et al. [37] proposed least squares support vector machine (LS-SVM) to improve SVM in the classification accuracy. To facilitate practical applications, LS-SVM has evolved from a binary classification method to a multi-classification one [38]. Hence, we employ LS-SVM for the fault diagnosis modelling. This is an intelligent method for the fault diagnosis of the 3D printer.

The rest of the paper is organized as follows: in Section 2, the transmission mechanism of the delta 3D printer is introduced to analyze different fault types. The attitude monitoring is subsequently proposed for diagnosing the printer faults. The data classification based on LS-SVM is also detailed in this section. In Section 3, the experiment on the fault diagnosis of delta 3D printer is carried out. The experimental results and discussions were given in Section 4. Peer methods are also compared in this section. Conclusions are drawn in Section 5.

## 2. Methodology

The transmission mechanism of the delta 3D printer is introduced in the first subsection. For better fault diagnosis of the printer, a novel method using the attitude monitoring is proposed. The next step is to use LS-SVM to generate a classification strategy for the data analysis. One can then obtain the health condition of the delta 3D printer.

### 2.1. Transmission Mechanism Analysis of the Delta 3D Printer

**Figure 1:**
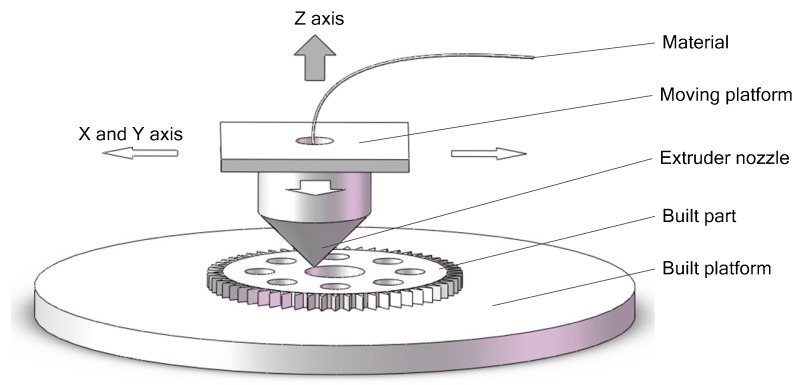
Schematic of the FDM process.

For 3D printing, laying down material in layers is the additive principle of fused deposition modeling (FDM) that was developed by Scott Crump in the late 1980s [39]. As described in Figure 1, a filament of the material is melted in a heated extruder nozzle fixed on a moving platform and deposited on a built platform. In company with the moving platform, the extruder nozzle is moved in the X-Y plane to print a layer of the product. Along the Z direction the moving platform moves up one step (the slice thickness) upon finishing this layer. The printer will repeat the aforementioned cycle for the next layer until the built part (i.e., printed product) is completed.

**Figure 2:**
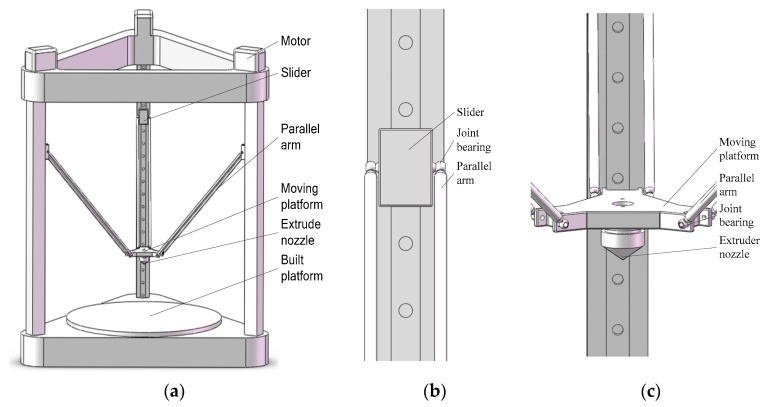
Typical structure of the delta 3D printer: (**a**) over view (**b**) local view of slider (**c**) local view of moving platform.

Based on the FDM theory, a typical delta 3D printer employs a symmetric structure for three parallel kinematic chains [40]. Referring to Figure 2a, the delta 3D printer is composed of a built platform, an extrusion nozzle fixed under a moving platform, six parallel arms and three sliders. For closed-loop parallel architectures, the number of degrees-of-freedom (DOFs) indicating how many variables to be controlled can be calculated by:

(1) $$F=\lambda (n-j-1)+\sum_{i}^{j}f_{i}-\sum_{i}^{j}p_{i},$$

where *F* represents the DOFs of the mechanism, λ is the DOFs of the space in which the mechanism works, *n* stands for the number of rigid bodies of the mechanism, *j* denotes the number of joints, *f_i_* is the DOFs of each joint, and $\sum_{i=1}^{j}p_{i}$ denotes the number of local DOFs and redundant constraints.

For the delta 3D printer, there are λ=6, n=11, j=15, $\sum_{i=1}^{j}f_{i}=39$ and $\sum_{i=1}^{j}p_{i}=6$. Substituting the aforementioned parameters into Equation (1) generates that there are three DOFs for the delta 3D printer. Thus, the delta 3D printer may be actuated by three electrical motors connected to three linear transmission units. In these parallel kinematic chains, in fact, each chain is a parallelogram which ensures that the moving platform is always parallel to the ground and also to the built platform. In other words, the fault appears when the moving platform with vibration in a certain range is not parallel to the built platform (except within the range of systematic error). This affects the printing quality and even leads to the failure of the 3D printing task.

A typical delta 3D printer has three vertical guides. Along each vertical guide a slider moves up and down, driven by a motor connected with a synchronous belt. The slider is connected to the moving platform by the parallel arm with a spherical joint, also known as a joint bearing. Local views of the slider and the moving platform are shown in Figure 2b,c, respectively. On the basis of geometric algorithms, it is known that the moving platform can be moved to any position in a cylindrical working space provided a corresponding position of the three sliders. 

**Figure 3:**
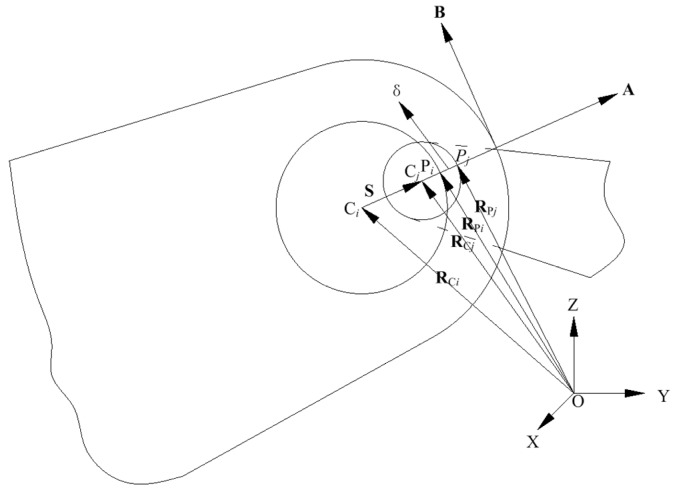
Contact kinematic of joint bearing with clearance.

According to the above fundamental analysis of the delta 3D printer, the wear and the clearance of the joint bearing may lead to a bias at the moving platform (i.e., a fault of the printer). Generally, kinematics, dynamics and wear models of a joint bearing with clearance can be established. With reference to Figure 3, the kinematic model of the joint with clearance can be expressed as [41]:

(2) $$S=R_{Cj}-R_{Ci},$$

where **S** is the eccentricity vector between the bearing and the ball with relative to global coordinate system, **R**_C*i*_ and **R**_C*j*_ are the position vector of C*_i_* and C*_j_* in global coordinate system, respectively. **R**_C*i*_ and **R**_C*j*_ can be rewritten as:

(3) $$\{\begin{array}{l} R_{Ci}=H_{Ci}t\\ R_{Cj}=r_{1}+R_{1}h_{Cj}^{1} \end{array},$$

where **H**_C*i*_ is the shape function, **t** is the nodal coordinates of moving platform, $r_{1}$ is the position vector between the origin of local coordinate system and the global coordinate system, and $h_{Cj}^{1}$ is the position vector of the center of gravity of the ball in the local coordinate system. The relationship between normal and tangential velocity is formulated as:

(4) $$\{\begin{array}{l} V_{A}=\left[{\left({\dot{R}}_{Pi}-{\dot{R}}_{Pj}\right)}^{T}A\right]A\\ V_{B}=\left({\dot{R}}_{Pi}-{\dot{R}}_{Pj}\right)-V_{A} \end{array},$$

where **V***_A_* is the normal velocity, and **V***_B_* is the tangential velocity.

To build the dynamics model of the delta parallel mechanism, the constraint equations among rigid bodies can be formulated as [42]:

(5) $$\left[\begin{array}{c} K(q^{0},q^{1}),K(q^{0},q^{2}),K(q^{0},q^{3}),K(q^{0},q^{4})\\ K(q^{6},q^{1}),K(q^{7},q^{2}),K(q^{8},q^{3}),K(q^{9},q^{4})\\ K(l,q^{7}),K(l,q^{8}),K(l,q^{9}),K(l,q^{5}),K(l,q^{5}) \end{array}\right]=0,$$

where **K**(*,#) represents the constraint equation between * and #, **q** denotes rigid cylinder body, and **l** stands for the moving platform. Then the dynamics model of delta parallel mechanism can be expressed as:

(6) $$\left[\begin{array}{ccc} N_{q} & 0 & K_{q}^{T}\\ 0 & N_{l} & K_{ql}^{T}\\ K_{q} & K_{ql} & 0 \end{array}\right]\left[\begin{array}{c} \ddot{q}\\ \ddot{l}\\ \tau \end{array}\right]=\left[\begin{array}{c} Q_{1}+Q_{2}\\ Q_{3}-Q_{4}\\ Q_{5} \end{array}\right],$$

where **N_q_** is the mass matrix of rigid bodies, **K_q_** is the Jacobian matrix of the kinematic constraint equations, **N_l_** is the mass matrix of flexible body, $\ddot{q}$ is the acceleration vector of rigid bodies, $\ddot{l}$ is the absolute acceleration vector of flexible body, τ is the Lagrange multipliers vector, **Q_1_** is the generalized external forces, **Q_2_** is quadratic velocity quadratic velocity including gyroscopic moment from differentiating the kinetic energy with respect to time and to the generalized coordinates, **Q_3_** is the elastic force of the finite element, **Q_4_** the generalized external nodal forces and contact force and **Q_5_** is the quadratic velocity.

The friction-induced wear model can be expressed by [11]:

(7) $$\frac{dU}{dE}=\mu D,$$

where *U* is the wear depth, *E* the sliding distance, *μ* the linear wear coefficient, and *D* the contact pressure.

Based on the above models, one can see that it is complicated and difficult to directly solve the fault problem of the delta 3D printer from the kinematics, dynamics and friction-induced wear models. Moreover, the above models are only for one joint. In a delta 3D printer, there are 12 joint bearings. Hence, in this work, a data-driven model is established for the diagnosis of the delta 3D printer to solve those problems.

In brief, the fault may result from the wear of the joint bearing. It is undetectable when the wear is less but it affects the quality of the printed products. To avoid the establishment and solution of the complicated kinematics, dynamics and friction-induced wear models, we try to use only one sensor to monitor the health condition of the delta 3D printer by taking into account kinematics, dynamics and friction-induced wear.

### 2.2. Data Collection in the Attitude Monitoring

According to [43], an attitude sensor can detect 3-axial attitude angle, angular velocity, vibratory acceleration and magnetic field intensity signals, which represent the parameters of the kinematics, dynamics and friction-induced wear models to a certain extent. Hence, an attitude sensor is employed to monitor the delta 3D printer in our work. The real-time attitude angle calculation can be solved by using the three-axial gyro, accelerometer and magnetometer separately [44]. With the gyro signal, one has:

(8) $$\theta_{i}=(\omega_{i}-\omega_{b})dt+\theta_{i-1},$$

where $\theta_{i}$ is the angle of the *i*-th time, $\omega_{i}$ is the angular velocity measured by the gyro at *i*-th time and $\omega_{b}$ is the angular velocity bias.

To calculate attitude angles, the measurements of three-axial accelerometer and three-axial magnetometer may be applied to the following equations:

(9) $$\begin{array}{c} \alpha =\tan^{-1}\left(\frac{a_{x}}{\sqrt{a_{y}^{2}+a_{z}^{2}}}\right),\beta =\tan^{-1}\left(\frac{a_{y}}{\sqrt{a_{x}^{2}+a_{z}^{2}}}\right),\\ \gamma =\tan^{-1}\left(\frac{m_{y}\cos\beta +m_{x}\sin\beta \sin\alpha -m_{z}\sin\beta \cos\alpha }{m_{x}\cos\alpha +m_{z}\sin\alpha }\right), \end{array}$$

where α, β and γ are pitch (the angle of rotation of X-axis), roll (the angle of rotation of Y-axis) and yaw (the angle of rotation of Z-axis), respectively; *a_x_*, *a_y_* and *a_z_* are X-axial, Y-axial and Z-axial acceleration, respectively; and *m_x_*, *m_y_* and *m_z_* are X-axial, Y-axial and Z-axial magnetic field intensity, respectively.

**Figure 4:**
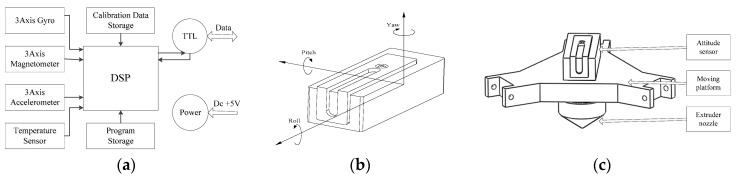
Attitude modelling theory: (**a**) the basic structure of the attitude sensor, (**b**) the reference frame and attitude angles, and (**c**) the schematic of the sensor installation.

Although the gyro has a very fast dynamic response, it has drift bias and may be affected by the temperature. Meanwhile, with the integral error accumulation, attitude angles calculated by gyro measurements will be inaccurate. Accelerometers are not applicable to dynamic measurement for the influence of acceleration motion. Magnetometers are sensitive to the interference of external magnetic fields. Hence, the attitude sensor based on MEMS and integrated with gyros, accelerometers and magnetometers is selected in this paper. As described in Figure 4a, the attitude sensor employs three-axial gyros assisted by three-axial accelerometers and three-axial magnetometers as well as compensated for the temperature. The gyro drifts in the pitch and roll are corrected by accelerometers. The correction of gyros drift in the yaw angle is performed by magnetometers. Then, using a Kalman filter for every sensing element [45], one can obtain the accurate attitude angles relative to the reference frame of the attitude sensor including pitch, roll and yaw as is shown in Figure 4b. By employing an attitude sensor, one can obtain not only three attitude angles, but also raw data of the three-axial gyro, three-axial accelerometer and three-axial magnetometer [46] filtered using finite impulse response (FIR) filter [47]. This means that there are twelve channels in the attitude monitoring. After a comprehensive consideration of the performance and price, the MEMS attitude sensor, whose price is about $400 US, was selected and all-channel data were used for fault diagnosis in this work.

Notice that the fault will be reflected by the attitude of the moving platform as long as the fault appears on the delta 3D printer. In addition, an attitude sensor can be made extremely light and compact by using MEMS technology. Any components in the transmission chain of the delta 3D printer may be faulty. However, it is uneconomical and unrealistic to install one sensor in every transmission chain component due to the limit of the installation space. For saving the number of sensors in a transmission chain, one can install a sensor at the end of the transmission chain to monitor the health condition of the whole transmission chain [33]. Hence, only one attitude sensor fixed on top of the moving platform is used as shown in Figure 4c. The connection between the moving platform and attitude sensor is a threaded connection to ensure that there is no relative movement. With the aid of the attitude sensor, one can easily obtain different attitude conditions of the moving platform, which reflects the health condition of the printer. Considering that the early wear of joint bearing is very weak, we employ LS-SVM to model the data so that the fault type of delta 3D printer can be obtained.

### 2.3. SVM Modelling in the Attitude Monitoring

After collecting attitude sensor data under different fault conditions, the next step is to generate a classification strategy. In this paper, SVM [48], one of the most precise classification algorithms in the field of supervised learning is utilized for the fault diagnosis modelling.

Aiming at diagnosing faults, health conditions of the delta 3D printer are divided into 13 patterns consisted of one normal and 12 faulty ones. In other words, it is a non-linear and multi-classification issue. The standard SVM is a binary classifier to define a hyperplane that separates the data from two different classes. As a modification version of the SVM, LS-SVM employs a least squares loss function and equality constraints for the classification.

Given a training set (*x_i_*, *y_i_*), where *x_i_* ($x_{i}\in R^{n}$) and *y_i_* ($y_{i}\in \{-1,\;+1\}$) represent the *i*-th (*i* = 1, 2, …, *k*) training sample and pattern, respectively. The objective function is formulated as [49]:

(10) $$\underset{w,b,e}{\min}\frac{1}{2}{\left\|w\right\|}^{2}+\frac{1}{2}\xi \sum_{i=1}^{k}e_{i}^{2},\;s.t.\;y_{i}[w^{T}\varphi (x_{i})+b]=1-e_{i},\;i=1,\;2,\;\ldots ,\;k,$$

where ξ is error penalty factor, **e***_i_* is the slack factor of **y***_i_*, **w** is the normal vector, φ(·) is nonlinear mapping function, and **b** is bias term. Lagrange form is defined as:

(11) $$L(w,b,e,\eta )=\frac{1}{2}{\left\|w\right\|}^{2}+\frac{1}{2}\xi \sum_{i=1}^{k}e_{i}^{2}-\sum_{i=1}^{k}\eta_{i}\{y_{i}[w^{T}\varphi (x_{i})+b]-1+e_{i}\},$$

where $\eta_{i}$ (*i* = 1, 2, …, *k*) are Lagrange multipliers. The optimal solution of the objective function is obtained, provided that partial derivatives of Lagrange function *L* is equal to zero with regard to **w**, **b**, **e** and η respectively. Then optimal solution can be expressed by the following linear equation:

(12) $$\left[\begin{array}{cc} 0 & -Y^{T}\\ Y & ZZ^{T}+\xi^{-1}I \end{array}\right]\left[\begin{array}{c} b\\ \eta \end{array}\right]=\left[\begin{array}{c} 0\\ \vec{1} \end{array}\right],$$

where Y = [**y**_1_; …; **y***_k_*], $Z=[\varphi {\left(x_{1}\right)}^{T}y_{1};\;\ldots ;\;\varphi {\left(x_{k}\right)}^{T}y_{k}]$, $\vec{1}=[1;\;\ldots ;\;1]$, $\eta =[\eta_{1};\;\ldots ;\;\eta_{k}]$. According to Mercer’s theorem, kernel function exists which corresponds to a dot product in a higher dimensional space. Accordingly, the decision function of LS-SVM is given by:

(13) $$f(x)=\mathrm{sgn}\left(\sum_{i=1}^{k}\eta_{i}y_{i}G(x_{i},x)+b\right),$$

where $G(x_{i},x)$ is the kernel function. In this work, radial basis function kernel is adopted below for non-linear data classification:

(14) $$G(x_{i},x_{j})=\exp\left(-\frac{{\left\|x_{i}-x_{j}\right\|}^{2}}{2\sigma^{2}}\right),$$

where σ is the kernel width.

The approaches of combination of binary classification, one-against-one and one-against-all, are usually applied to multi-classification [50]. The method of one-against-one, which constructs *N*(*N* − 1)/2 classifiers for *N*-class issue (i.e., each pair of classes has one binary classifier), is adopted to complete multi-fault pattern recognition in this work. The testing sample *x* is classified by voting in the following form:

(15) $$\{\begin{array}{ll} V_{i}=V_{i}+1; & f_{ij}(x)=1\\ V_{j}=V_{j}+1; & f_{ij}(x)=-1 \end{array},\;i=1,\;\ldots ,\;N-1;\;j=i+1,\;\ldots ,\;N,$$

where *V_k_* is the votes of *k*-class (*k* = 1, …, *N*), the original value of *V_k_* equal to zero, and *f_ij_*(*x*) is the classifier between *i*-class (positive) and *j*-class (negative). If *V_c_* is the maximum when all binary classifiers completed the voting, then testing sample *x* is assigned to *c*-class. To avoid over fitting problem, cross-validation algorithm was adopted to optimize parameters in this work [51,52].

There are two ways for applying the attitude sensor data in samples generation. In Equation (16), only the optimal channel may be used. On the contrary, another feasible solution is to employ all channels of the sensor as shown in Equation (17):

(16) $$x=g_{1}(d_{opt}),$$

(17) $$x=g_{2}(d_{1},d_{2},\;\ldots ,\;d_{12}),$$

where *x* is the training or testing sample, *d_opt_* is the optimal channel that makes the highest classification among twelve channels, *d_i_* is the *i*-th channel (*i* = 1, …, 12). In our work, we first employ Equation (16) for the attitude monitoring, while applying Equation (17) for comparison.

### 2.4. Overview of the Present Attitude Monitoring with SVM

**Figure 5:**
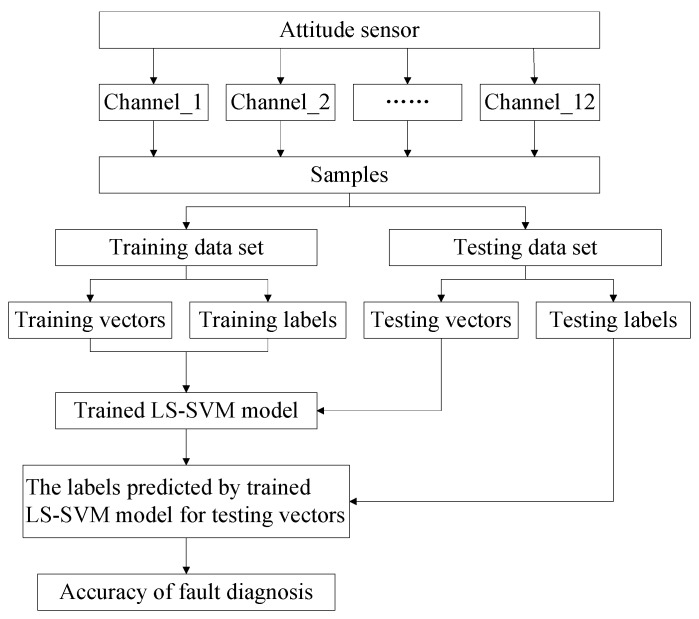
Overview of the proposed approach.

According to the above analysis, Figure 5 illustrates the schematic of the proposed approach, which is also summarized as below.

*Step 1*. Collect data from the attitude sensor installed on the moving platform of the delta 3D printer in different faulty types;
*Step 2*. All channels data are employed to generate training and testing samples with given labels;
*Step 3*. Train LS-SVM model;
*Step 4*. Test trained LS-SVM model;
*Step 5*. Output the labels (health condition of the delta 3D printer) predicted by the trained LS-SVM model;
*Step 6*. Compare predicted labels with testing labels; and
*Step 7*. Output the fault diagnosis accuracy. End.

## 3. Experiments

**Figure 6:**
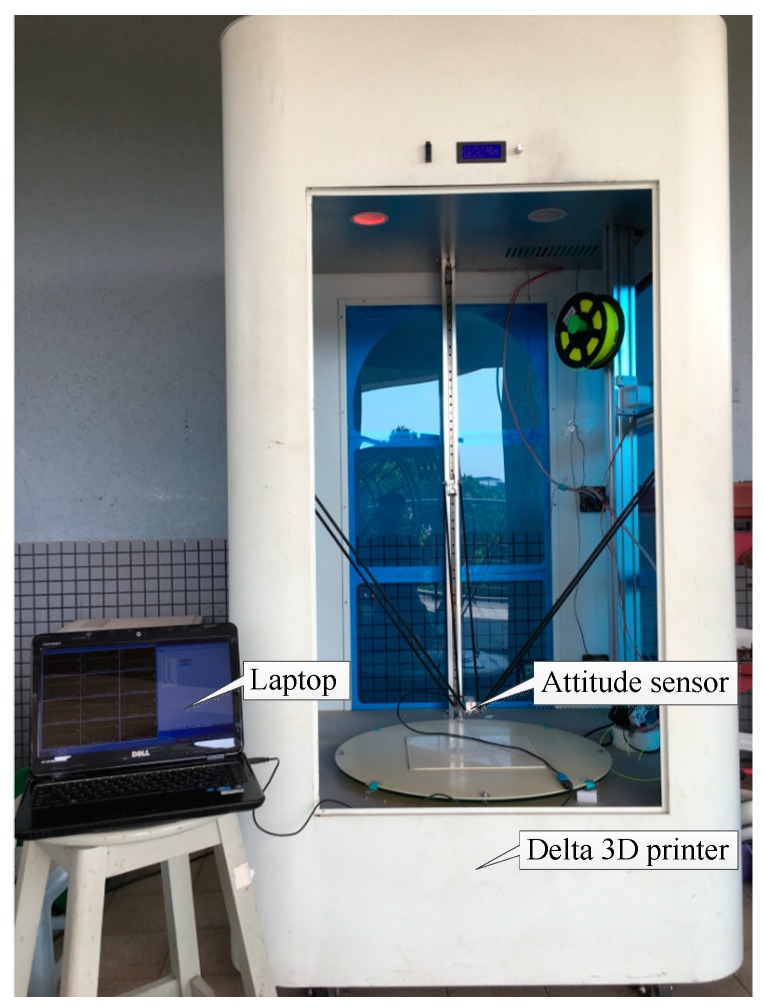
Experimental configurations for the fault diagnosis of the delta 3D printer.

To validate the effectiveness of the present method, an experimental setup was built as shown in Figure 6. In the experiments, a delta 3D printer (SLD-BL600-6, SHILEIDI, Dongguan, China), an attitude sensor (AH100B, RION, Shenzhen, China) and a laptop (Inspiron N4110, DELL, Round Rock, DX, USA) were connected together. Resolutions of attitude angle, angular velocity, vibratory acceleration and magnetic field intensity are less than 0.1°, 0.1°/s, 0.098 m/s^2^ and 0.25 μT, respectively. The delta 3D printer was used to provide a predefined movement at the moving platform. Different abrasions were pre-planted on the joint bearing. The attitude sensor was mounted on the moving platform to collect condition data under different fault conditions. The laptop offers G-codes for the delta 3D printer through Repetier-Host (a slicing software) and collects the output data from the attitude sensor via an upper monitor, where the calibration of the attitude sensor, the observation of real-time data and the adjustment of the sampling frequency can be implemented. In this work, sampling frequency was set at 100 Hz.

In our scheme, the wear of a joint bearing was set as a faulty pattern. As introduced in Section 2.1, a typical delta 3D printer has 12 joint bearings (named as A, B, …, and L, respectively). In the experiments, the normal condition was label as pattern No. 1. Different faulty patterns on each joint bearing were labeled as pattern No. 2, No. 3, …, and No. 13, respectively. All the 13 condition patterns are listed in Table 1. Considering that connections between sliders, moving platform and joint bearings are of thread, the screw (0.7 mm pitch) of each joint bearing was loosened a half-turn to simulate a fault (i.e., 0.35 mm clearance each).

**Figure 7:**
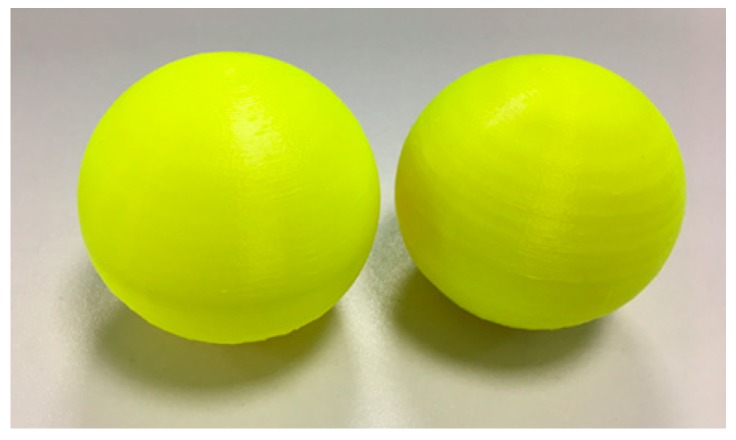
Printed balls with the normal condition (**left**) and the faulty joint bearing A (**right**).

Before data collection, two balls were printed by the delta 3D printer used in this experiment under the normal condition (pattern No. 1) and the faulty joint bearing A (pattern No. 2). As shown in Figure 7, the quality of printed ball was affected by the health condition. If the fault can be detected by using the proposed approach, one may avoid the waste of materials.

In the experiments, a cylindrical shell model with radius 75 mm and height 0.3 mm was first established in a 3D modeling software, and then was saved as a STL format file. The G-codes for the trail (ten circles with radius 75 mm) of moving platform were generated by importing the STL file into the slicing software. In this way, the theoretical movement of the moving platform is ten circles with radius of 75 mm on the same layer in one experiment. Moving a circle with radius 75 mm takes moving platform 21.3 s in the experiments. Each experiment was repeated three times for collecting the data using the aforementioned program. In other words, there are 30 circle data in each condition pattern. In each condition pattern, 300 samples were obtained by evenly dividing each circle into ten pieces. Finally, 3900 samples were obtained in total. In each data sample, there are 12 channels each of which has 213 data acquisition points. All samples were randomly sorted before training and testing of LS-SVM model. In each data analysis, 2730 (70%) samples were used for model training, while the rest 1170 (30%) samples for the fault diagnosis testing.

## 4. Results and Discussion

### 4.1. Fault Diagnosis Results Using the Proposed Method

Using all channels data as a sample, the LS-SVM modelling and testing were repeated 6 times. The highest accuracy is 95.56% and the lowest one is 93.42%. The mean value of the fault diagnosis accuracy is 94.44% with variance 0.00007101. The overall accuracy of each time is provided in Table 2.

### 4.2. Comparison with Peer Methods

#### 4.2.1. LS-SVM Modelling with Only One Channel

For comparison, we also employed only one of the twelve channels for LS-SVM modelling. The fault diagnosis results are shown in Table 3. Higher accuracy (about 71.40%) was obtained when the data of No. 10, 11 or 12 channels (X-axial, Y-axial or Z-axial magnetic field intensity) were used for LS-SVM modelling. On the other hand, the classification is worse (about 6.78%) using the data of No. 7, 8 or 9 channel (X-axial, Y-axial or Z-axial acceleration). This means that the solely application of vibration acceleration is insufficient for the fault diagnosis of the delta 3D printer. In addition, the highest classification accuracy (73.68%) in this method is inferior to all channels data as shown in Table 2.

#### 4.2.2. BPNN Modelling with Data from One of the Twelve Channels

The same data was used for BPNN modeling. Table 4 shows the fault diagnosis results using BPNN modelling with each channel data of the attitude sensor. The highest fault diagnosis accuracy is 56.71% which is inferior to the results as shown in Table 3. This indicates that LS-SVM is superior to BPNN in the fault diagnosis modelling.

#### 4.2.3. BPNN Modelling with All the Twelve Channels Data

The performances using all channels data are presented in Table 5. Compared to Table 2 and Table 3, it is shown that LS-SVM modelling with all channels of the attitude sensor features the highest fault diagnosis accuracy for the delta 3D printer.

## 5. Conclusions

The printing quality is dramatically affected by the health condition of the 3D printer. In this paper, an attitude monitoring method with SVM has been proposed for the fault diagnosis of the delta 3D printer. By collecting the monitoring data in different condition patterns, the attitude samples were applied for SVM modelling via supervised learning for model training and testing. The highest accuracy (94.44%) in this study has been achieved while all channel data was utilized for fault diagnosis with LS-SVM modelling. For comparison, only one channel data were used for SVM modelling. The results show that different channel data could lead to different fault diagnosis accuracy levels. However, only using one channel data is insufficient for the fault diagnosis of the delta 3D printer. On the other hand, BPNN model has been built using the same data as well. Unfortunately, performances of BPNN models are unable to meet the requirement of fault diagnosis regardless of using optimal channel or all channels.

## Figures and Tables

**Table 1:** Condition patterns set in the experiments.

Pattern No.	Description of the Delta 3D Printer
1	Normal
2	Faulty joint bearing A
3	Faulty joint bearing B
4	Faulty joint bearing C
5	Faulty joint bearing D
6	Faulty joint bearing E
7	Faulty joint bearing F
8	Faulty joint bearing G
9	Faulty joint bearing H
10	Faulty joint bearing I
11	Faulty joint bearing J
12	Faulty joint bearing K
13	Faulty joint bearing L

**Table 2:** Fault diagnosis results using LS-SVM model with all channels data.

Channel	Repeat Order						Mean (%)	Variance
	1(%)	2(%)	3(%)	4(%)	5(%)	6(%)		
All channels	94.79	95.56	93.50	94.96	94.44	93.42	94.44	0.00007101

**Table 3:** Fault diagnosis results using LS-SVM model with one of the twelve channels data.

Channel	Repeat Order						Mean (%)	Variance
	1(%)	2(%)	3(%)	4(%)	5(%)	6(%)		
1	35.64	35.56	36.75	33.08	36.50	35.98	35.59	0.00017258
2	31.71	30.94	33.68	31.37	34.10	30.51	32.05	0.00022082
3	67.86	67.35	70.43	68.21	68.21	67.95	68.34	0.00011529
4	45.04	44.36	41.97	42.91	43.08	41.97	43.22	0.00015705
5	37.52	39.83	37.52	39.91	36.75	40.60	38.69	0.00025875
6	33.16	31.03	31.54	32.91	32.99	32.74	32.40	0.00007836
7	6.24	6.50	6.15	7.01	5.64	5.90	6.24	0.00002288
8	6.58	5.81	6.15	6.50	6.84	8.80	6.78	0.00011080
9	8.55	6.32	6.84	6.75	6.84	6.24	6.92	0.00007042
10	71.37	72.14	72.05	71.45	69.57	73.68	71.71	0.00017888
11	71.20	72.05	71.11	70.68	71.37	71.97	71.40	0.00002781
12	71.71	69.66	72.22	72.48	73.16	68.89	71.35	0.00028694

**Table 4:** Fault diagnosis results using BPNN model with all the twelve channels data.

Channel	Repeat Order						Mean (%)	Variance
	1(%)	2(%)	3(%)	4(%)	5(%)	6(%)		
1	25.21	20.68	17.86	9.57	27.95	23.42	20.78	0.00424408
2	26.58	22.99	23.59	22.82	23.25	20.43	23.28	0.00038810
3	54.96	56.84	55.04	50.51	48.72	56.50	53.76	0.00112073
4	15.04	18.89	14.27	15.98	17.26	18.97	16.74	0.00038868
5	17.26	15.64	15.90	15.21	15.38	14.79	15.70	0.00007295
6	13.42	15.81	13.33	15.81	16.32	14.36	14.84	0.00017198
7	25.56	25.30	15.98	25.73	28.63	23.33	24.09	0.00186552
8	33.76	31.37	37.26	33.93	31.71	30.43	33.08	0.00060961
9	10.77	12.31	11.45	11.26	8.29	12.39	11.08	0.00022557
10	57.69	49.23	57.26	50.68	59.83	60.68	55.90	0.00230168
11	54.62	58.12	57.35	54.02	58.21	57.95	56.71	0.00035579
12	36.41	37.18	34.62	37.69	30.60	32.99	34.92	0.00074956

**Table 5:** Fault diagnosis results using BPNN model with all the twelve channels data.

Channel	Repeat Order						Mean (%)	Variance
	1(%)	2(%)	3(%)	4(%)	5(%)	6(%)		
All channels	49.40	50.85	12.48	43.85	45.85	9.49	35.34	0.03629351
